# OPA1 Enhances Microglial Amyloid-β Clearance and Alleviates Cognitive Impairments in an Alzheimer’s Disease Model

**DOI:** 10.14336/AD.2025.0082

**Published:** 2025-04-15

**Authors:** Qing Wang, Mengqi Dong, Xue Xia, Xinyu Bao, Mengsha Hu, Lei Ye, Yun Xu

**Affiliations:** ^1^Department of Neurology, Nanjing Drum Tower Hospital Clinical College of Nanjing Medical University, Nanjing 210008, China.; ^2^Department of Neurology, Nanjing Drum Tower Hospital, Affiliated Hospital of Medical School, Nanjing University, Nanjing 210008, China.; ^3^Department of Neurology, Nanjing Drum Tower Hospital Clinical College of Nanjing University of Chinese Medicine, Nanjing 210008, China.; ^4^Department of Neurology, Nanjing Drum Tower Hospital, State Key Laboratory of Pharmaceutical Biotechnology and Institute of Translational Medicine for Brain Critical Diseases, Nanjing University, Nanjing 210008, China.; ^5^Jiangsu Key Laboratory for Molecular Medicine, Medical School of Nanjing University, Nanjing 210008, China.; ^6^Jiangsu Provincial Key Discipline of Neurology, Nanjing 210008, China.; ^7^Nanjing Neurology Medical Center, Nanjing 210008, China.; ^8^Nanjing Neuropsychiatry Clinic Medical Center, Nanjing 210008, China.

**Keywords:** OPA1, microglial amyloid-β clearance, cognitive impairments, Alzheimer’s disease

## Abstract

Amyloid deposition is thought to be a pathologic hallmark of Alzheimer disease (AD), which is associated with cognitive decline. Microglia play a crucial role in the pathology of AD, especially in the clearance of Aβ. Optic atrophy 1 (OPA1) is a GTPase primarily on the inner mitochondrial membrane, related to mitochondrial dynamics and cellular energy metabolism. Here, we found that decreased OPA1 expression and defective mitochondrial morphology in microglia during AD. Next, we utilized an OPA1 activator BGP-15, an OPA1 inhibitor myls22 and an OPA1 overexpression virus to investigate the role of OPA1 in AD. Our findings demonstrate that OPA1 promotes ATP production and Aβ clearance by microglia, leading to improved cognitive function. This may relate to down-regulation of hexokinase-2 (HK2) expression. These results suggest a critical role for OPA1 in Aβ clearance by microglia and a promising new direction for therapeutic approaches in AD.

## INTRODUCTION

Alzheimer’s disease (AD), the most common neurodegenerative disease, is characterized by progressive cognitive impairment and stands as the leading cause of dementia [1]. The primary pathology of AD is deposition of β-amyloid (Aβ) plaques within the brain, which is closely associated with cognitive impairment in AD [2]. Microglia, the resident immune cells of the central nervous system (CNS), rapidly initiate immune responses, and play a pivotal role in modulating neuroinflammation and eliminating various pathological proteins including the phagocytosis of amyloid-β (Aβ) deposits [3]. A recent study based on human brain tissues have confirmed that in AD patients receiving either active (AN1792) or passive (Lecanemab) immunotherapy, microglia mediate Aβ clearance through the triggering receptor expressed on myeloid cells 2 (TREM2)-apolipoprotein E (APOE) signaling axis. Notably, immune activation induces a metabolic shift in microglia from glycolysis to oxidative phosphorylation, enhancing mitochondrial ATP production and microglial phagocytic function [4]. However, metabolic deficiencies within microglia impair their phagocytic capacity, exacerbating Aβ accumulation and contributing to disease progression in AD [5-8]. In animal models, the deletion of translocator protein (TSPO) in microglia led to compromised mitochondrial respiration and enhanced mitochondrial hexokinase-2 (HK2) recruitment, triggering a metabolic shift to glycolysis, which in turn impaired phagocytic activity [7]. Consequently, enhancing the mitochondrial energy metabolism in microglia may represent a novel therapeutic strategy for AD.

Mitochondria are essential cellular organelles, responsible for energy production and numerous metabolic functions [9]. Optic atrophy 1 (OPA1), a key protein in mitochondrial dynamics, plays a pivotal role in maintaining mitochondrial morphology and cristae integrity [10, 11]. OPA1 facilitates the fusion of mitochondrial inner membranes, ensuring the efficient distribution of mitochondrial DNA (mtDNA), metabolites, and other essential components throughout the mitochondrial network, thus maintaining mitochondrial homeostasis and functional integrity [12]. In a rat model of Middle cerebral artery occlusion/reperfusion (MCAO/R), downregulation of OPA1 during ischemic-reperfusion injury impairs mitochondrial fusion, leading to neuronal death, whereas upregulation of OPA1 during reperfusion restores mitochondrial architecture and alleviates neuronal loss [13].

Beyond its role in mitochondrial fusion, OPA1 independently regulates mitochondrial cristae structure and density. OPA1 deficiency both in OPA1 knockout mouse embryonic fibroblasts(MEFs) [14] and conditional OPA1-deleted mice [15], induced significant mitochondrial structural abnormalities, impairing oxidative phosphorylation and adenosine triphosphate (ATP) production. Additionally, OPA1 modulates apoptosis by regulating cytochrome c release [16]. Furthermore, dysregulation of OPA1 has been linked to microglia activation, neuroinflammation and cognitive dysfunction, particularly in diseases such as retinal ischemia-reperfusion injury and intracerebral hemorrhage [17, 18]. However, the role of OPA1 in microglia during AD remains unclear.

In this study, we examined the alterations in OPA1 expression in hippocampal microglia of APPswe/PSEN1dE9 (APP/PS1) mice and elucidated the mechanistic role of OPA1 in regulating cognitive function through microglia-mediated Aβ clearance. Our findings reveal a previously unrecognized function of OPA1 in Aβ clearance, providing novel insights into potential therapeutic avenues for Alzheimer’s disease.

## MATERIALS AND METHODS

### Mouse models and treatment

Male APP/PS1 mice (7 months old) and age-matched wild-type mice were obtained from Gempharmatech Co., Ltd (Nanjing, China). Mice were treated daily with 20mg/kg BGP-15 dissolved in 10% dimethyl sulfoxide (DMSO) or vehicle 10% DMSO (S8370, Selleck, Shanghai, China) for 15 censecutive days by oral gavage. For virus injection into the hippocampal region, mice were anesthetized and placed in a stereotaxic frame. OPA1 overexpression adenovirus or control adenovirus were injected into the CA1 at the following coordinates relative to bregma: AP:-1.82, L:±1.13, DV:-1.25. Bilateral injections of 1μl were carried out using a 10μl microliter syringe (Gaoge, Shanghai, China). Animals were housed in group of maximum eight in individually ventilated cages under standard conditions (22°C,12h light-dark cycle) receiving food and water ad libitum. All animal experimental procedures related to animal care and treatment were met the guidelines of the National Animal Care and Use Committee and approved by the Institutional Animal Care and Use Committee (IACUC) of Nanjing University.

### Postmortem Samples

Human brain tissue was provided by the Chinese Brain Bank Center (CBBC, http://cbbc.scuec.edu.cn) (Wuhan, China). The use of human brain tissues was approved by the Research Ethics Committee of South-Central University for Nationalities (2021-scuec-034).

### Primary microglial culture

Primary microglia were isolated from astroglia/microglia mixed cultures derived from newborn C57BL/6 pups as described previously [19]. Floating cells were isolated by shaking the flask after 10 days in culture and seeded onto 12-well plates for 12h. Primary cultured microglia were treated with human Aβ_1-42_ (2μM; Millipore, Darmstadt, Germany) prepared as previous described [20] for 24h, myls22 (30μM; MCE, USA) for 48h or BGP-15 (100μM; S8370, Selleck, Shanghai, China) for 24h. Primary cultured microglia were infected with either the opa1-overexpression lentivirus or its control lentivirus (with a multiplicity of infection of 10) purchased from PSTE biotechnology (Nanjing, China). ATP level was assessed by an ATP assay kit (S0026, Beyotime, Shanghai, China) according to the manufacturer’s instructions.

### Behavioral experiments

#### Open field

To evaluate locomotor activity and anxiety-like behavior, we performed an open field test. The open field test was performed in a 48cm×48cm×36cm arena which was divided into 16 squares of equal area. Each test mouse was placed in the center and allowed to explore the open filed box for 10min. The mean speed and time spent in the center and corner zone were recorded and quantified by ANY-maze software (Stoelting, USA). After each mouse test, the apparatus was thoroughly disinfected with 75% alcohol to eliminate any residual odors and prevent interference with subsequent experimental results.

#### New object recognition (NOR)

A novel object recognition (NOR) test took advantage of mice’s innate preference for novelty to reveal the recognition memory of mice. Prior to testing, mice are placed in an open field measuring 48cm×48cm×36cm for 3 consecutive days to acclimatize to the environment. Then two identical objects were placed in the middle of the box during the 10-min training session and the mice were observed to explore the objects. After a half-hour interval, one of the objects was replaced with a different-shaped object, and the mice were allowed to freely explore for 5 min again. The time spent exploring new object and the total time spent exploring objects were counted separately and the discrimination index was calculated to evaluate the memory ability of mice.

#### Morris water maze

The Morris water maze (MWM) test was used to test the learning and memory ability of mice for spatial location. The experimental setup consisted of a circular water tank filled with clear water, maintained at a temperature of 20-22°C. An appropriate amount of titanium dioxide (TiO2) was added to the water to create a milky appearance, thereby concealing the location of the hidden platform. The mice were placed in the water in four different areas and allowed to explore for 1 min for 5 consecutive days where a hidden platform under the water. If the mice do not find the platform within the stipulated time, they would be placed on the platform for 1 min to familiarize themselves with the platform. On the sixth day of the test, the platform was removed, and the mice were allowed to swim freely in the pool for 60 s. The swimming speed, number of platform crossings, escape latency, and time spent within the target quadrant were recorded using ANY-maze software.

#### Y maze

The Y-maze consists of three closed arms (named A, B, C), and the mice were uniformly placed at the distal end of the A arm and allowed to explore freely for 8 min. All arm entries and the number of alternations were recorded to assess the short-term memory of mice.

### Quantitative real-time PCR

Total RNA was extracted by a TRIzol reagent kit (Invitrogen, USA) according to the standard protocol. The PrimeScript RT Reagent kit (Takara) was used to reversely transcribe the extracted total RNA into cDNA. Quantitative PCR analysis was performed using an ABI 7500 PCR instrument (Applied Biosystems) with the SYBR Green PCR kit (Takara). The relative expression levels of each gene shown were normalized to β-actin (Table 1).

**Table 1:** The primers used were as follows.

Gene	Primer
**Actin**	F: ACGGCCAGGTCATCACTATT R: TGGCATAGAGGTCTTTACGGA
**OPA1**	F: TGGAAAATGGTTCGAGAGTCAG R: CATTCCGTCTCTAGGTTAAAGCG
**HK2**	F: TGATCGCCTGCTTATTCACGG R: AACCGCCTAGAAATCTCCAGA

### Western blotting

The cell samples were discarded from the culture plate and washed three times with pre-chilled phosphate buffered saline (PBS). They were then lysed on ice for 30 mins with RIPA (P0013B, Beyotime) buffer plus protease inhibitor (HY-K0022, MCE), transferred to EP tubes and centrifuged at 14,000 rpm for 25 minutes at 4°C. The mouse hippocampal tissue was dissected after perfusion with PBS and lysed with the previously mentioned protein lysis buffer on ice for 30 minutes, followed by centrifugation at 14,000 rpm for 25 minutes at 4°C. The supernatant from both the cells and tissue was collected and the protein concentration was measured using the BCA Assay (P0012, Beyotime). Subsequently, proteins were separated by 10% sodium dodecyl sulfate polyacrylamide gel electrophoresis (SDS-PAGE) and blotted onto a 0.2mm PVDF membrane. Unspecific bindings were blocked with 5% non-fat milk in Tris buffered saline with 0.1% Tween-20(TBST) for 2h at room temperature and membranes were then incubated with some primary antibody overnight at 4°C. The following primary antibodies and dilutions were used: rabbit anti-OPA1 (1:1000; bioworlde, 27733-1-ap), rabbit anti-HK2 (1:1000, abclonal, A22319), mouse anti β-actin (1:1000, sigma, A5441). Secondary HRP-conjugated goat anti-rabbit antibody at 1:5000 dilution (beyotime, a0423) or HRP-conjugated goat anti-mouse antibody at 1:5000 dilution (beyotime, a0428) was incubated for 2h at room temperature. Secondary antibodies were visualized using the SuperFemto ECL Chemiluminescence Kit (E423, Vazyme), and the gray value of protein bands was analyzed and quantified using ImageJ software.

### Immunofluorescence staining

Brain tissue was fixed in 4% paraformaldehyde (PFA) for 24h at 4 °C, while cells were fixed for 15 min with 4% PFA. Following fixation, the brains were sequentially dehydrated in 15% and 30% sucrose solutions. The brains were then embedded in OCT and collected at 20-μm-thick serial coronal slices using a cryostat (Thermo, CryoStar NX50). The samples were punctured with PBS containing 0.25% triton X-100 (PBST) and blocked with 2% bovine serum albumin (BSA) at room temperature for 2 hours to reduce nonspecific antibody binding. The samples were incubated with the following primary antibody 4 °C overnight: goat anti-IBA1 (1:500, Abcam, ab5076), rabbit anti-OPA1 (1:100; bioworlde, 27733-1-ap), mouse anti- amyloid beta antibody (6E10) (1:200; BioLegend, 803014), Rat anti-CD68 (1:500, Abcam, ab53444). The following day, the wet boxes were removed from the refrigerator and allowed to equilibrate at room temperature for 30 minutes. Subsequently, the sections were incubated with secondary antibodies (Invitrogen, 1:500) in the dark at room temperature for 2 h and counterstained with DAPI (1:5000, Bioworlde, Louis Park, MN, USA) for 10 min. To exclude non-specific background staining, secondary antibody-only controls were performed in parallel by omitting the primary antibodies during the staining procedure. No detectable fluorescence signal was observed in these control sections, confirming the specificity of the immunolabeling. The samples were washed three times with PBS and mounted using an anti-fade mounting medium (P0126, beyotime). The images were captured using a fluorescence microscope (Olympus IX73) or confocal laser-scanning microscope (Olympus FV3000) and analyzed with Image J software.

### Analyses of mitochondrial morphology

Electron microscopy was used to evaluate the ultrastructure of mitochondria in the microglia of hippocampal from mice. To visualize mitochondrial morphology of primary microglia, we performed MitoTracker Green staining (M7514, Invitrogen, USA) according to the manufacturer’s protocol. Mitochondria parameters were analyzed using Image J software (NIH, Bethesda, MD, USA).

### Phagocytosis assays

The primary microglia were cultured with Aβ_1-42_ (HiLyte™ Fluor 488; AS-60479-01, Anaspec, Fremont, CA) to analyse the Aβ_1-42_ phagocytosis of cell. Aβ_1-42_ was aggregated at 37°C for 5 days before use. After different treatments, Aβ_1-42_ (0.9μM) was added to the cells and incubated at 37°C for 3-4h. The medium was removed, and cells were washed with serum-free Dulbecco's Modified Eagle Medium (DMEM) to remove excess of Aβ_1-42_. Phagocytic activity of microglia was examined using flow cytometric analysis and immunofluorescence staining. For flow cytometric analysis, the cells were digested by trypsin for 5 min and the digestion was terminated by DMEM containing 10% FBS. After centrifugation at 800 rpm for 5 min at 4 °C, the supernatant was aspirated, and cells were resuspended in pre-cooled PBS. The flow cytometric analysis was carried out with BD Accuri C6 Flow Cytometer (BD Biosciences) analyzed using FlowJo v 10 (Tree Star, Ashland, OR). For immunofluorescence staining, the samples were incubated with goat anti-IBA1 and rat anti-CD68 as previously described. Images were processed and analyzed by Image J software.

### Electrophysiology

Mice were sacrificed by anesthetized with 1.25% Avertin and fresh hippocampal slices (300 μm) were prepared as described previously [19]. Slices were incubated in oxygenated artificial cerebrospinal fluid (ACSF) for at least 2h before recordings. The MEA-2100-60-System (Multi Channel Systems, Reutlingen, Germany) was used to record Field excitatory postsynaptic potential (fEPSP) in the stratum radiatum of the hippocampal CA1 region. Input-output curves were obtained by recording the fEPSP slope with increasing stimulator intensity (from 0 to 100 μA). For long-term potentiation (LTP) experiments, the stimulation intensity was normally set at 50% of maximum response amplitude. In LTP recordings, after 30 min of stable baseline, LTP was induced by using the high-frequency stimulation protocol (HFS; 100 Hz, 1s duration, 10s). The fEPSP slopes were normalized by the averaged slope value over the 30-min baseline, and slopes in the last 5 min of recordings were averaged. The data were collected and analyzed with LTP-Director software and LTP-Analyzer software.

### Statistical analysis

All data analysis used by GraphPad Prism 8.0 software (GraphPad Prism, Boston, MA, USA). Data were tested for normality using the Shapiro-Wilk test prior to analysis. For normally distributed data, results were presented as mean ± SEM from at least 3 independent experiments. An unpaired Student's t test was used to compare differences between two groups and one-way or two-way analysis of variance (ANOVA) with Bonferroni or Tukey’s post hoc test was used for statistical difference between more than two groups. For non-normally distributed data, results were expressed as the interquartile range. Differences between two groups were compared using the Mann-Whitney U test, whereas comparisons among more than two groups were analyzed using the Kruskal-Wallis rank sum test with Dunn's correction. Statistical differences were considered significant for values of p<0.05.

**Figure 1.**
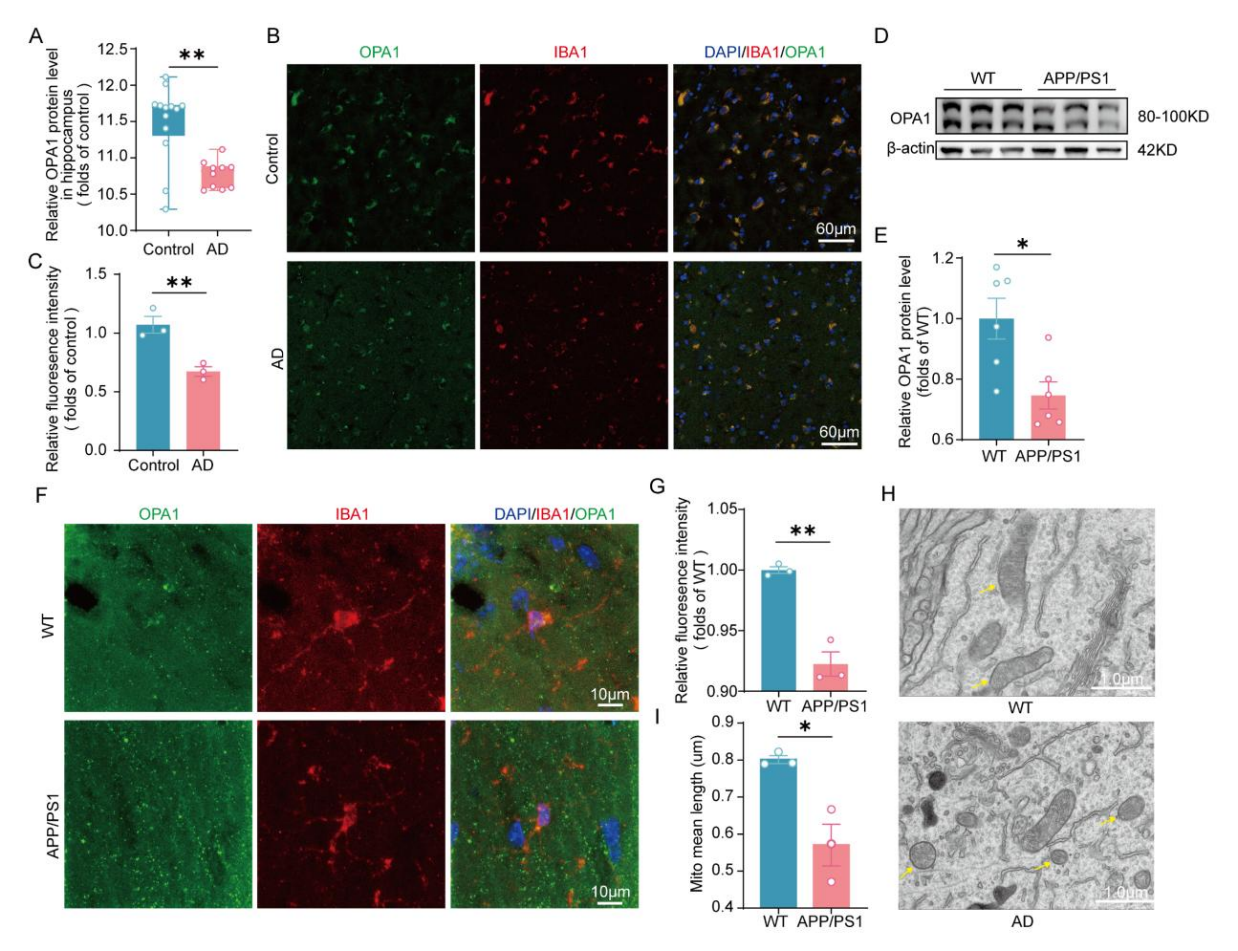
**Diminished OPA1 expression and impaired mitochondrial morphology in microglia during AD**. (**A**) The expression of OPA1 in the hippocampus of control and AD patients from the alzdata database. n = 10-13 for each group. p=0.0041. (B) Lower levels of OPA1 (green) immunoreactivity in IBA-1+ (red) microglia were detected in the temporal cortex of AD group compared to control group. (**C**) Quantitative analysis of colocalization of IBA-1 and OPA1 in control and AD patients. n = 3 for each group. *t*(4) = 4.843, p=0.0084. (**D**) Representative western blot image of OPA1 in the hippocampus of WT and APP/PS1 mice. (**E**) Western blot analysis of OPA1 in the hippocampus of WT and APP/PS1 mice. β-Actin was used as a loading control. n = 6 for each group. t(10) = 3.131, p=0.0107. (F) Lower levels of OPA1 (green) immunoreactivity in IBA-1^+^ (red) microglia were detected in APP/PS1 mice. (**G**) Quantitative analysis of colocalization of IBA-1 and OPA1 in WT and APP/PS1 mice. n = 3 for each group. *t*(4) =7.464, p=0.0017 (H and I) Electron microscopic (EM) images (H) and quantitative measures (I) of microglial mitochondria in the hippocampus of APP/PS1 mice. n=3 for each group. *t*(4) =4.017, p=0.0159. Mann-Whitney U test for (A), Unpaired two-tailed test for (C), (E), (G), and (I). *p < 0.05, **p < 0.01.

## RESULTS

### Diminished OPA1 expression and impaired mitochondrial morphology in microglia during Alzheimer’s disease.

First, we found that the expression of OPA1 was decreased in the hippocampus of AD patients by consulting the databases (Fig. 1A) (www.alzdata.org/). Furthermore, we examined colocalization of microglia with OPA1 by immunostaining for IBA1 (microglia marker) and OPA1. Our immunostaining results showed OPA1 expression in microglia decreased in the temporal cortex of AD patients compared to normal individuals (Fig. 1B-1C). Similarly, OPA1 protein levels were significantly lower in the hippocampus of APP/PS1 mice compared to wild type (WT) mice (Fig. 1D-1E). Besides, immunostaining showed a similar result in the hippocampus of APP/PS1 mice (Fig. 1F-1G). We performed electron microscopy to visualize the mitochondrial morphology of microglia in the hippocampus of 8-month-old APP/PS1 mice and found that microglial mitochondria in the hippocampus of APP/PS1 mice were significantly shorter than those in WT group (Fig. 1H-1I).

**Figure 2.**
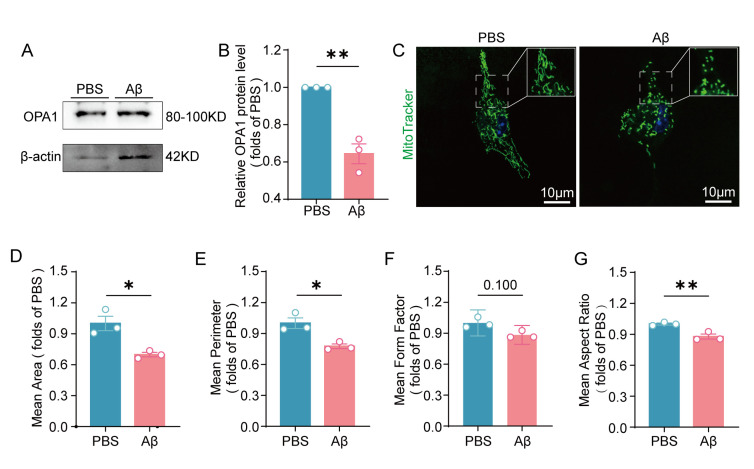
**Treatment of Aβ_1-42_ resulted in decreased OPA1 expression and mitochondrial fragmentation**. (**A**) Representative western blot image of OPA1 in the primary microglia and Aβ_1-42_-treated primary microglia. (**B**) Western blot analysis of OPA1 in the primary microglia and Aβ_1-42_-treated primary microglia. β-Actin was used as a loading control. n=3 for each group. *t*(4)=6.732, p=0.0025. (**C**) Representative images of MitoTracker Green fluorescence in the primary microglia and Aβ_1-42_-treated primary microglia. (**D-G**) Mitochondrial morphology assessment including mean area, perimeter, mean form factor and mean aspect ratio. n=3 for each group. n=3 for each group. *t*(4)=4.142, p=0.0144 in (D), *t*(4)=3.988, p=0.0163 in (E); p=0.100 in (F), *t*(4)=4.698, p=0.0093 in (G). Unpaired two-tailed test for (B), (D), (E), and (G). Mann-Whitney U test for (F), *p < 0.05, **p < 0.01.

To further assess mitochondrial morphology of microglia in AD, primary microglia were treated with Aβ_1-42_. First, we found that the protein levels of OPA1 in microglia were significantly reduced after Aβ_1-42_ treatment (Fig. 2A-2B). Mitochondria staining with Mitotracker Green revealed that Aβ_1-42_ treatment led to smaller mitochondrial size and altered shape in microglia, as shown by decreased mean area, perimeter, form factor, and aspect ratio (Fig. 2C-2G). In summary, these results revealed decreased OPA1 expression and impaired mitochondrial morphology of microglia in AD.

### Overexpression or Activation of OPA1 improved cognitive function of APP/PS1 mice

**Figure 3.**
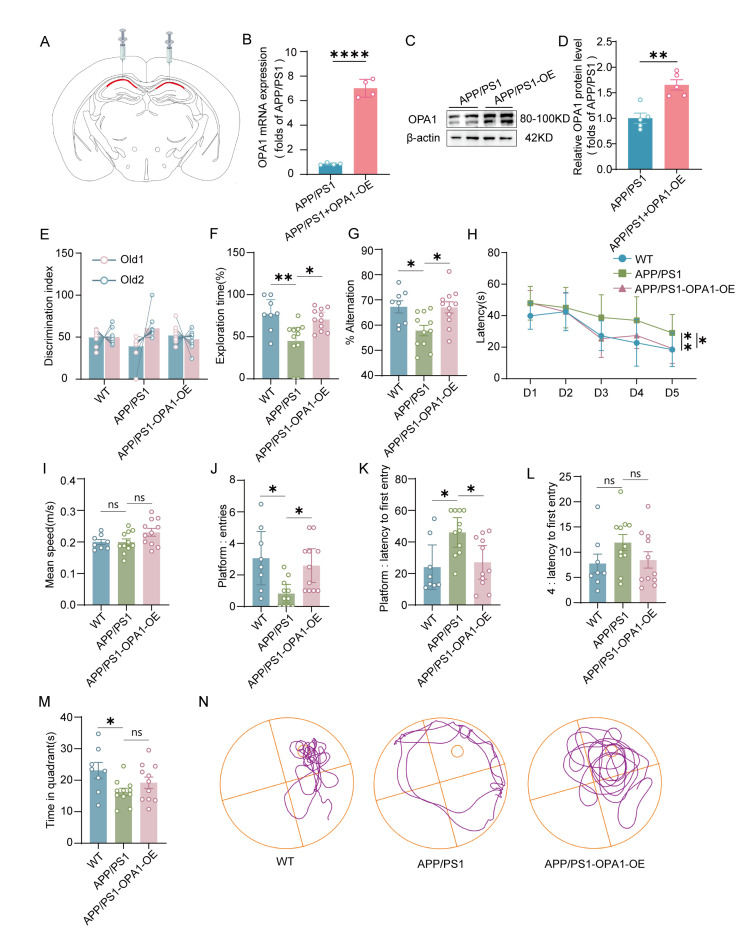
**OPA1 overexpression enhanced cognitive function in APP/PS1 mice**. (**A**) Schematic of adenovirus microinjection into the CA1. (**B**) The level of OPA1 was measured by qPCR and normalized to β-actin mRNA. *t*(6)=16.76, p <0.0001. (**C**) Representative western blot image of OPA1 in the hippocampus of APP/PS1 and APP/PS1-OPA1-OE mice. (**D**) Western blot analysis of OPA1 in the hippocampus of APP/PS1 and APP/PS1-OPA1-OE mice. β-Actin was used as a loading control. n=5 for each group. t(8)=16.76, p=0.0018. (**E-F**) The time spent exploring two identical objects during the training process (E) and the novel object during the test session (F) by WT, APP/PS1 and APP/PS1-OPA1-OE mice in the NOR test was recorded. n=8-11 for each group. APP/PS1 versus WT group: p =0.0073, APP/PS1 versus APP/PS1-OPA1-OE group: p =0.0353. (**G**) The percentage of spontaneous alternation by WT, APP/PS1 and APP/PS1-OPA1-OE mice in the Y maze test was scored. n=8-11 for each group. F(2,27)=0.0236, APP/PS1 versus WT group: p =0.0156, APP/PS1 versus APP/PS1-OPA1-OE group: p =0.0155. (**H**)The escape latency of WT, APP/PS1 and APP/PS1-OPA1-OE mice was analyzed in the training process. n=8-11 for each group. F(2,27)=4.876, APP/PS1 versus WT group: p =0.0061, APP/PS1 versus APP/PS1-OPA1-OE group: p =0.0297. (**I-M**) The swimming speed (I), number of platform crossings (J), latency to the platform (K), latency to the target quadrant (L) and time spent in the target quadrant (M) of WT, APP/PS1 and APP/PS1-OPA1-OE mice were recorded in the probe trial. n=8-11 for each group. *F*(2,27)=1.414, APP/PS1 versus WT group: p >0.9999, APP/PS1 versus APP/PS1-OPA1-OE group: p =0.1269 for swimming speed; APP/PS1 versus WT group: p =0.0166, APP/PS1 versus APP/PS1-OPA1-OE group: p =0.0207 for the number of platform crossings. APP/PS1 versus WT group: p =0.0177, APP/PS1 versus APP/PS1-OPA1-OE group: p =0.0327 for latency to platform. *F*(2,27)=0.1521, APP/PS1 versus WT group: p =0.3150, APP/PS1 versus APP/PS1-OPA1-OE group: p =0.4241 for latency to the target quadrant. *F*(2,27)=1.714, APP/PS1 versus WT group: p =0.0425, APP/PS1 versus APP/PS1-OPA1-OE group: p =0.6944 for time in the target quadrant. (**N**) Representative moving trajectory of WT, APP/PS1 and APP/PS1-OPA1 OE mice. Unpaired two-tailed test for (B) and (D). One-way ANOVA followed by Bonferroni post hoc test for (G), (I), (L), and (M). Two-way ANOVA followed by Tukey’s post hoc test for (H). Kruskal-Wallis test followed by Dunn's post-hoc correction for (F), (J) and (K). *p < 0.05, **p < 0.01, ***p < 0.001, ****p < 0.0001, ns no significance.

Based on the above observations, we injected an OPA1-overexpressing adenovirus(OPA1-OE) or a control adenovirus into the CA1 of 6-month-old APP/PS1 mice (Fig. 3A-3B). One month after stereotactic injection, we verified the upregulation of OPA1 expression in the CA1 following viral infection using RT-PCR and Western blot (Fig. 3C-3E). We performed behavioral tests to assess cognitive function in mice. In the open field test, there were no significant differences in the movement speed and time spent in the center or corner zone in each group of mice, which suggested similar locomotion or anxiety-related behaviors (Supplementary Fig. 1A-1D). In the NOR task, APP/PS1-OPA1-OE mice exhibited significantly increased exploration time for the novel object compared to APP/PS1 mice, indicating that OPA1 overexpression effectively enhances object recognition memory in APP/PS1 mice (Fig. 3F-3G). In the Y-maze test, APP/PS1-OPA1-OE mice showed a significantly higher spontaneous alternation rate, suggesting improved short-term spatial working memory (Fig. 3H). In the MWM test, APP/PS1 mice demonstrated a significantly prolonged escape latency compared to WT mice, whereas APP/PS1-OPA1-OE mice showed a markedly reduced escape latency compared to APP/PS1 mice (Fig. 3I). Similarly, no significant differences in swimming speed were observed among the groups (Fig. 3J).

In the probe test, APP/PS1-OPA1-OE mice exhibited not only a shorter escape latency but also a higher number of platform crossings compared to APP/PS1 mice (Fig. 3K-3O). The above results suggested that OPA1 overexpression attenuated learning and memory impairment in AD mice.

**Figure 4.**
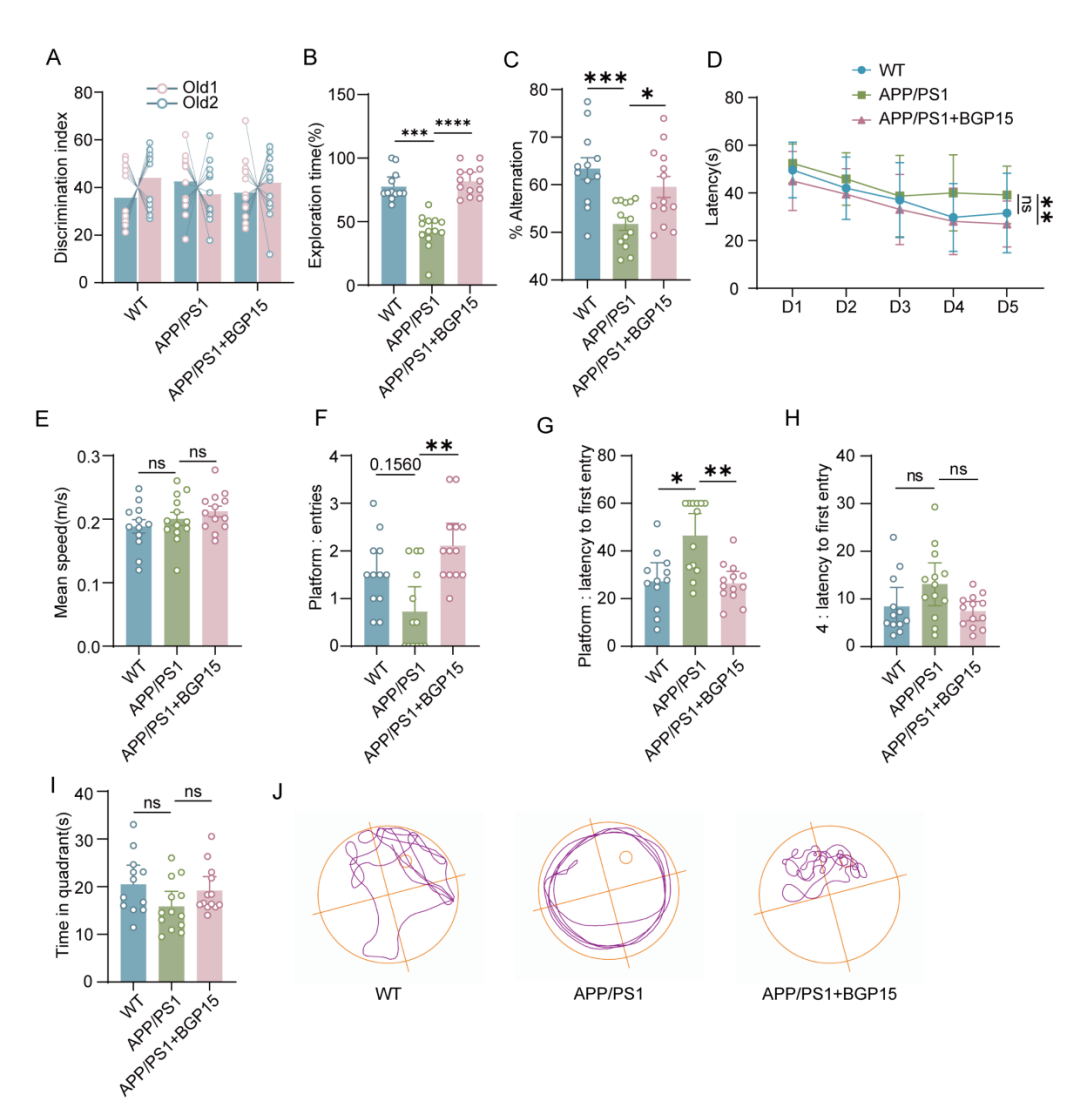
**OPA1 activation enhanced cognitive function in APP/PS1 mice**. (A and B) The time spent exploring two identical objects during the training process (A) and the novel object during the test session (B) by WT, APP/PS1 and BGP-15-treated APP/PS1 mice in the NOR test was recorded. n=12-13 for each group. APP/PS1 versus WT group: p =0.004, APP/PS1 versus APP/PS1+BGP15 group: p <0.0001. (**C**) The percentage of spontaneous alternation by WT, APP/PS1 and BGP-15-treated APP/PS1 mice in the Y maze test was scored. n=12-13 for each group. F(2,35)=0.9722, APP/PS1 versus WT group: p =0.0006, APP/PS1 versus APP/PS1+BGP15 group: p =0.0228. (**D**) The escape latency of WT, APP/PS1 and BGP-15-treated APP/PS1 mice was analyzed in the training process. n=12-13 for each group. F(2,35)=3.469, APP/PS1 versus WT group: p =0.1247, APP/PS1 versus APP/PS1+BGP15 group: p =0.0015. (**E-I**) The swimming speed (E), number of platform crossings (F), latency to the platform (G), latency to the target quadrant (H) and time spent in the target quadrant (I) of WT, APP/PS1 and BGP-15-treated APP/PS1 mice were recorded in the probe trial. n=12-13 for each group. *F*(2,35)=0.1077, APP/PS1 versus WT group: p >0.9999, APP/PS1 versus APP/PS1+BGP15 group: p >0.9999 for swimming speed; APP/PS1 versus WT group: p =0.1560, APP/PS1 versus APP/PS1+BGP15 group: p =0.0015 for the number of platform crossings. APP/PS1 versus WT group: p =0.0175, APP/PS1 versus APP/PS1+BGP15 group: p =0.0047 for latency to platform. APP/PS1 versus WT group: p =0.1563, APP/PS1 versus APP/PS1+BGP15 group: p =0.1168 for latency to the target quadrant. APP/PS1 versus WT group: p =0.1015, APP/PS1 versus APP/PS1+BGP15 group: p =0.1954 for time in the target quadrant. (**M**) Representative moving trajectory of WT, APP/PS1 and BGP-15-treated APP/PS1 mice. One-way ANOVA followed by Bonferroni post hoc test for (C) and (E). Two-way ANOVA followed by Tukey’s post hoc test for (D). Kruskal-Wallis test followed by Dunn's post-hoc correction for (B), (F), (G), (H)and (I). *p < 0.05, **p < 0.01, ***p < 0.001, ****p < 0.0001, ns no significance.

Subsequently, we investigated the effects of OPA1 on memory functions with an OPA1 activator BGP-15 [21]. First, we performed behavioral tests to examine the impact of BGP-15 on cognitive deficits in APP/PS1 mice. Open field tests showed no significant differences in locomotor activity or anxiety behavior across the groups (Fig. S1E-S1H). In the NOR task, APP/PS1 mice treated with BGP-15 spent more time exploring the novel object, exhibiting significantly improved performance relative to that of APP/PS1 mice treated with DMSO (Fig. 4A-4B). In the Y-maze test, APP/PS1 mice treated with BGP-15 were more likely than APP/PS1 mice treated with DMSO to explore alternate arms (Fig. 4C). In addition, the APP/PS1 mice took longer latency time to find hidden platform compared to the WT group, while the BGP-15-treated APP/PS1 mice showed reduced latency time (Fig. 4D). There were no significant differences in swimming speed in each group of mice, while BGP-15-treated APP/PS1 mice exhibited a significant decrease escape latency and increased crossing times compared with APP/PS1 mice treated with DMSO (Fig. 4E-4I). These results indicated that activation of OPA1 alleviated cognitive dysfunction in APP/PS1 mice.

### OPA1 activation or overexpression improved Aβ clearance of microglia and synaptic plasticity

**Figure 5.**
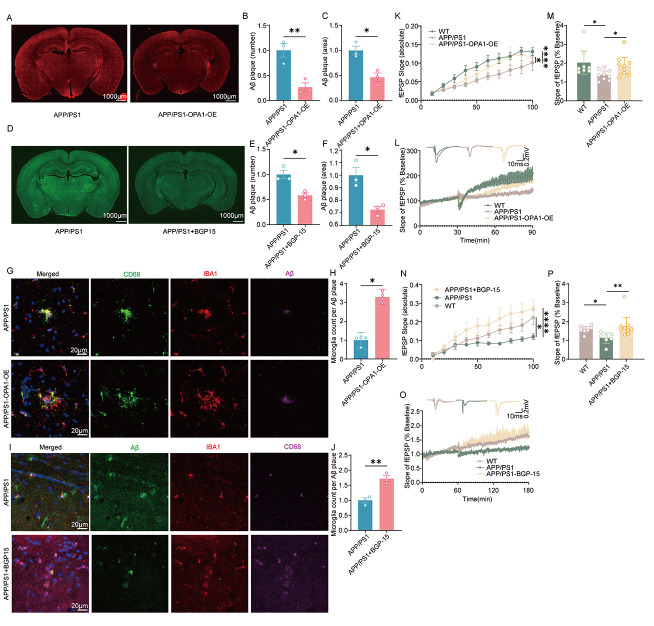
**OPA1 overexpression or activation improved Aβ clearance of microglia and synaptic plasticity (A) Representative images showing the Aβ (red) deposition in the hippocampus of APP/PS1 and APP/PS1-OPA1-OE mice**. (**B-C**) Aβ plaque counts and areas in hippocampus were low in APP/PS1-OPA1-OE mice compared with APP/PS1 mice. n = 3 for each group. t(4) = 4.621 p=0.0099 in (B), t(4) = 4.376 p=0.0119 in (C). (**D**) Representative images showing the Aβ (green) deposition in the hippocampus of APP/PS1 and BGP-15-treated APP/PS1 mice. (**E-F**) Aβ plaque counts and areas in hippocampus were low in BGP-15-treated APP/PS1 mice compared with APP/PS1 mice treated with DMSO. n = 3 for each group. t(4) = 4.330 p=0.0123 in (E), t(4) = 4.167, p=0.0141 in (F). (**G**) Representative pictures showing colocalization of Aβ (magenta) with IBA-1 (red) and CD68 (green) in the hippocampus of WT, APP/PS1 and APP/PS1-OPA1-OE mice. (**H**) The number of microglia surrounding Aβ was higher in APP/PS1-OPA1-OE mice compared with APP/PS1 mice. n = 4 for each group. p=0.0286 (I)Representative pictures showing colocalization of Aβ (green) with IBA-1 (red) and CD68 (magenta) in the hippocampus of WT, APP/PS1 and BGP-15-treated APP/PS1 mice. (**J**) The number of microglia surrounding Aβ was higher in BGP-15-treated APP/PS1 mice compared with APP/PS1 mice. n = 3 for each group. *t*(4) = 5.483, p=0.0054 (K) The input-output response increased in APP/PS1-OPA1-OE mice (n=3 slices/mouse, N=3 mice/group) compared with APP/PS1 mice (n=3 slices/mouse, N=3 mice/group). F(2,22)=2.035, APP/PS1 versus WT group: p <0.0001, APP/PS1 versus APP/PS1-OPA1-OE group: p=0.0399. (L and M) The slope of the evoked fEPSP increased in APP/PS1-OPA1-OE mice (n=3slices/mouse, N=3mice/group) compared with APP/PS1 mice during LTP induction (n=3slices/mouse, N=3mice/group). APP/PS1 versus WT group: p=0.0262, APP/PS1 versus APP/PS1-OPA1-OE group: p=0.416. (**N**) The input-output response increased in BGP-15-treated APP/PS1 mice (n=2-4 slices/mouse, N=3 mice/group) compared with APP/PS1 mice treated with DMSO (n=2-4 slices/mouse, N=3 mice/group). F(2,20)=4.289, APP/PS1 versus WT group: p =0.0260, APP/PS1 versus APP/PS1+BGP15 group: p <0.0001. (O and P) The slope of the evoked fEPSP increased in BGP-15-treated APP/PS1 mice (n=3slices/mouse, N=3mice/group) compared with APP/PS1 mice treated with DMSO during LTP induction (n=3slices/mouse, N=3mice/group). APP/PS1 versus WT group: p =0.0103, APP/PS1 versus APP/PS1+BGP15 group: p=0.0063. Unpaired two-tailed test for (B), (C), (E), (F)and(J). Mann-Whitney U test for (H), Two-way ANOVA followed by Tukey’s post hoc test for (K) and (N). Kruskal-Wallis followed by Dunn's post-hoc test for (M)and (P). *p < 0.05, **p < 0.01, ***p < 0.001, ****p < 0.0001

We next performed immunostaining to assess microglial phagocytosis of Aβ and found reduced Aβ deposition in APP/PS1-OPA1-OE mice or APP/PS1 mice treated with BGP-15 (Fig. 5A-5F). Furthermore, we utilized IBA1 and CD68 to label phagocytic microglia and the confocal images confirmed that IBA-1^+^/CD68^+^ phagocytic microglia showed increased engulfment of Aβ in the hippocampus of APP/PS1-OPA1-OE mice or BGP-15-treated APP/PS1 mice (Fig. 5G-5L).

Studies have shown that the accumulation of Aβ affects the synapse function [22, 23]. We next conducted electrophysiological recordings to examine synaptic transmission and synaptic plasticity. The slope of fEPSP increased with stimulus intensity in the hippocampus of APP/PS1-OPA1-OE mice compared with APP/PS1 mice. A similar trend was also observed in APP/PS1 mice treated with BGP15 compared with APP/PS1 mice treated with DMSO, which indicates improved synaptic transmission (Fig. 5E and 5P). Furthermore, OPA1-OE adenoviral injection or BGP-15 treatment resulted in higher averages of normalized fEPSP slope following LTP induction than APP/PS1 mice injection with control adenovirus or treated with DMSO (Fig. 5N-5O and 5Q-5R). Taken together, these results confirmed the positive effects of OPA1 on Aβ clearance and synaptic plasticity during AD.

### OPA1 regulated ATP production and Aβ clearance by microglia

**Figure 6.**
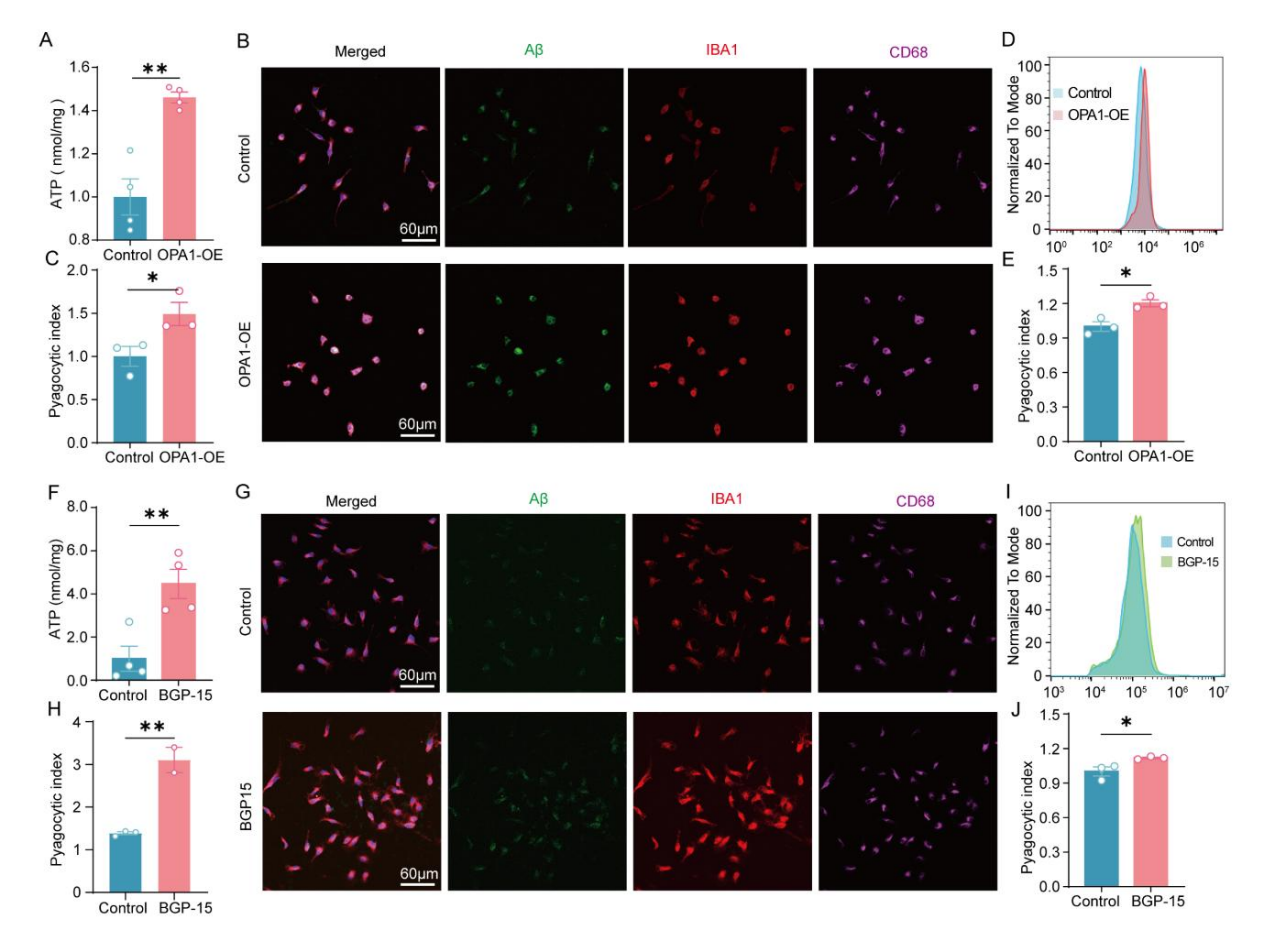
**Overexpression or activation of OPA1 contributed to increased ATP content and Aβ clearance by microglia**. (**A**) Total cellular ATP measured using an ATP assay kit (Beyotime) and normalized against total protein concentration. n=4 for each group. *t*(6)=4.181, p=0.0019. (**B**) Colocalization of Aβ (green) with IBA-1 (red) and CD68 (magenta) in the control and OPA1-OE group. (**C**) Quantitative analysis of phagocytic Aβ in the control and OPA1-OE group. n=3 for each group. *t*(4)=2.781, p=0.0498. (D and E) Flow cytometric analysis (D) and quantification (E) of microglial phagocytic activity for HiLyte™ Fluor 488-labeled Aβ_1-42_. n=3 for each group. *t*(4)=3.917, p=0.0173. (**F**) Total cellular ATP measured using an ATP assay kit (Beyotime) and normalized against total protein concentration. n=4 for each group. *t*(6)=3.907, p=0.0079. (**G**) Colocalization of Aβ (green) with IBA-1 (red) and CD68 (magenta) in the control and BGP-15-treated group. (**H**) Quantitative analysis of phagocytic Aβ in the control and BGP-15-treated group. n=2-3 for each group. *t*(3)=7.598, p=0.0047. (I and J) Flow cytometric analysis (I) and quantification (J) of microglial phagocytic activity for HiLyte™ Fluor 488-labeled Aβ_1-42_. n=3 for each group. *t*(4)=2.952, p=0.0419. Unpaired two-tailed test for (A), (C), (E), (F), (H) and (J). *p < 0.05, **p < 0.01.

The efficiency of microglial phagocytosis of Aβ plaques relies on the energy supply of mitochondria [6-8]. Given the energy-dependent nature of microglial phagocytosis [24], we explored whether microglial OPA1 modulates Aβ clearance through energy generation. Primary microglia were transfected with BGP-15 or an OPA1 overexpression (OPA1-OE) lentivirus. The stable transfection efficiency was verified via mCherry fluorescence and qPCR (Supplementary Fig. 2A and 2B). The ATP content of microglia was measured using ATP Assay Kit, confirming that activation or overexpression of OPA1 restored the ATP content compared to the control group (Fig. 6A and 6F).

HiLyte™ Fluor 488-labeled Aβ_1-42_ was added to cells after treatment, and microglia phagocytosis experiment revealed that activation (Fig. 4B-4E) or overexpression of OPA1 in microglia correlated with increased Aβ_1-42_ engulfment in vitro (Fig. 6G-6J).

**Figure 7.**
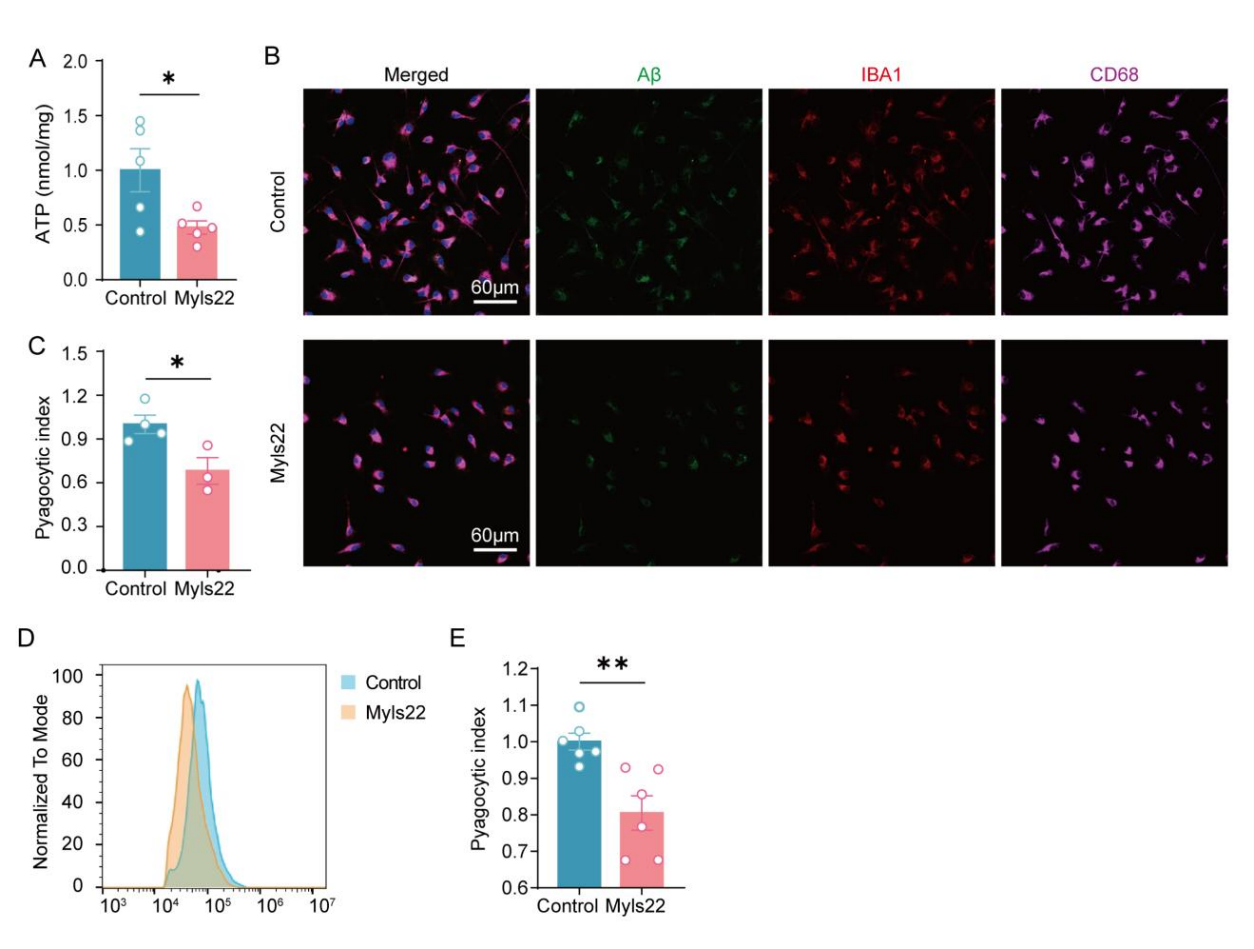
**OPA1 Inhibition reduced ATP content and Aβ clearance by microglia**. (**A**) Total cellular ATP measured using an ATP assay kit (Beyotime) and normalized against total protein concentration. n=5 for each group. *t*(8)=2.551, p=0.0341. (**B**) Colocalization of Aβ (green) with IBA-1 (red) and CD68 (magenta) in the control and myls22-treated group. (**C**) Quantitative analysis of phagocytic Aβ in the control and myls22-treated group. n=3-4 for each group. *t*(5)=2.981, p=0.0308. (D and E) Flow cytometric analysis (D) and quantification (E) of microglial phagocytic activity for HiLyte™ Fluor 488-labeled Aβ_1-42_. n=6 for each group. *t*(10)=3.708, p=0.0041. Unpaired two-tailed test for (A), (C), and (E). *p < 0.05, **p < 0.01.

In addition, microglia were treated with the OPA1 inhibitor myls22 (30 μM; MCE, USA) to assess Aβ clearance. The results revealed that myls22 treatment led to a significant decrease in ATP content (Fig. 7A). The control and myls22-treated microglia were cultured with HiLyte™ Fluor 488-labeled Aβ_1-42_, and the phagocytic index was evaluated using flow cytometric analysis and immunofluorescence staining. Our data suggested that the inhibition of OPA1 significantly reduced the phagocytic index of microglia (Fig. 7B-7E).

### Decreased expression of HK2 potentially involved in OPA1-mediated Aβ clearance by microglia

**Figure 8.**
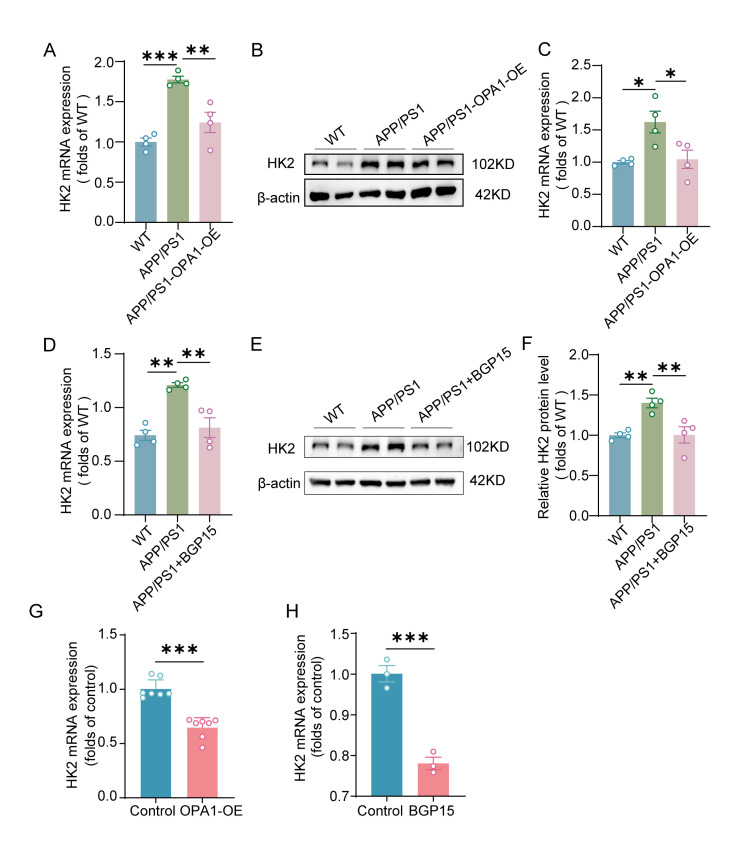
**Downregulated HK2 expression was associated with OPA1-mediated microglial Aβ clearance**. (**A**) The level of HK2 was measured by qPCR and normalized to β-actin mRNA. n=4 for each group. *F*(2,9)=1.523, APP/PS1 versus WT group: p =0.0003, APP/PS1 versus APP/PS1-OPA1-OE group: p=0.0039. (**B**) Representative western blot image of HK2 in the hippocampus of WT, APP/PS1 and APP/PS1-OPA1-OE mice. (**C**) Western blot analysis of HK2 in the hippocampus of WT, APP/PS1 and APP/PS1-OPA1-OE mice. β-Actin was used as a loading control. n=4 for each group. *F*(2,9)=3.938, APP/PS1 versus WT group: p =0.0221, APP/PS1 versus APP/PS1-OPA1-OE group: p=0.0330. (**D**) The level of HK2 was measured by qPCR and normalized to β-actin mRNA. n=4 for each group. F(2,9)=17.31, APP/PS1 versus WT group: p =0.0012, APP/PS1 versus APP/PS1+BGP15 group: p=0.0036. (**E**) Representative western blot image of HK2 in the hippocampus of WT, APP/PS1 and BGP-15-treated APP/PS1 mice. (**F**) Western blot analysis of HK2 in the hippocampus of WT, APP/PS1 and BGP-15-treated APP/PS1 mice. β-Actin was used as a loading control. n=4 for each group. *F*(2,9)=0.8322, APP/PS1 versus WT group: p =0.0080, APP/PS1 versus APP/PS1+BGP15 group: p=0.0086. (**H-I**) The mRNA level of HK2 in the primary microglia in the control and OPA1-OE group (H) or in the control and myls22-treated group (I) was measured by qPCR and normalized to β-actin mRNA. n=7 for each group. p=0.0006 in (D). n=3 for each group. *t*(4)=8.701, p=0.0010 in (E). One- way ANOVA followed by Bonferroni post hoc correction test for (A), (C), (D) and (F). Unpaired two-tailed test for (G) and (H). *p < 0.05, **p < 0.01, ***p < 0.001.

HK2 plays a pivotal role in mitochondrial energy metabolism and has been implicated in the clearance of amyloid-beta (Aβ) [7, 8]. Thus, we investigated whether opa1-mediated Aβ clearance was related to HK2 expression level. Our results showed that the expression level of HK2 was significantly upregulated in the hippocampus of APP/PS1-OPA1-OE mice but downregulated in the hippocampus of APP/PS1 mice (Fig. 8A-8C). Similarly, the expression level of HK2 was markedly upregulated in the hippocampus of DMSO-treated APP/PS1 mice, whereas it was downregulated in the hippocampus of APP/PS1 mice treated with BGP-15 (Fig. 8D-8F). Overexpression or activation of OPA1 also reduced the expression of HK2 in microglia (Fig. 8G-8H). This suggested that decreased expression of HK2 might be associated with opa1-mediated Aβ clearance.

## DISCUSSION

In this study, we confirmed that OPA1 activation could enhance Aβ clearance by microglia and improve cognitive functions in APP/PS1 mice. The OPA1 expression was decreased in microglia from both AD patients and AD mouse models. Additionally, treatment of Aβ_1-42_ also markedly reduced the level of OPA1 in primary microglia. Furthermore, OPA1 modulates ATP production and Aβ clearance by microglia, potentially through the downregulation of HK2 expression. Thus, our findings indicate a potential role for OPA1 in microglial clearance of Aβ and propose a new idea for enhancing Aβ clearance in AD.

OPA1, a GTPase with kinetic associations, is localized to the inner mitochondrial membrane, where it plays a crucial role in promoting mitochondrial fusion and maintaining mitochondrial morphology [25]. In addition, Opa1-dependent mitochondrial cristae remodeling could influence the assembly and activity of the respiratory chain complexes, which are essential for energy metabolism [14]. Mutations in OPA1 have been implicated in various diseases, with the most common being autosomal dominant optic atrophy (ADOA) and dominant optic atrophy plus syndrome (DOA+), the latter of which is characterized by multisystem neurodegeneration including myopathy, ataxia, and dementia.

ADOA is linked to several pathogenic mutations, including missense mutations in the GTPase domain [26, 27], frameshift mutations leading to truncated proteins (e.g., c.2708_2711delTTAG) [28, 29], and splice-site mutations disrupting mRNA splicing (e.g., IVS8+4C>T) [30]. Haploinsufficiency is considered the most common pathogenic mechanism, as OPA1 is a dosage-sensitive gene and the presence of only one functional allele is inadequate to sustain normal mitochondrial dynamics and function, especially in retinal ganglion cells that exhibit exceptionally high energy requirements. Missense mutations in the GTPase domain are classified as dominant negative effects, which interfere with the function of the wild-type protein, disrupt the mitochondrial fusion (mitochondrial fragmentation) and lead to more severe phenotypes, such as DOA+. Although rare, biallelic mutations in OPA1 have been linked to severe early-onset phenotypes, including Behr syndrome [31], Leigh-like stroke syndromes [32]and fatal infantile mitochondrial encephalomyopathy [33]. These findings underscore the critical role of OPA1 in the pathophysiology of various central nervous system diseases, including those associated with dementia-like symptoms.

Previous studies have shown that OPA1 expression declines in AD patients and transgenic AD mouse model, and OPA1 overexpression can effectively alleviate Aβ_1-42_-induced mitochondrial damage and neuronal apoptosis [34-36]. In addition, tau pathology has been shown to compromise mitochondrial dynamics by downregulation OPA1, which could result in the mitochondrial dysfunction in AD [37]. In our study, we also observed reduced OPA1 expression in AD patients and APP/PS1 mice and found that OPA1 activation could improve cognitive impairment in AD mice.

In the context of neurodegeneration and dementia, OPA1 mutations may impair mitochondrial fusion, cristae integrity, and energy metabolism, contributing to synaptic dysfunction and neuronal loss. Studies have demonstrated that OPA1 downregulation or deficiency disrupts synaptic stability, in cultured primary neurons in vitro [38] and in the hippocampal CA1 region of OPA1^+/-^ mice by downregulating key synaptic proteins such as postsynaptic density protein-95(PSD95) [39]. Additionally, OPA1 deletion in pro-opiomelanocortin (POMC) neurons disrupted mitochondrial fusion and cristae organization, leading to electron transport chain dysfunction, and ATP depletion [40]. Moreover, OPA1 deficiency promotes cytochrome c release and activates the caspase cascade, culminating in neuronal apoptosis. In contrast, it has been shown that OPA1 overexpression effectively preserves mitochondrial integrity prevents cytochrome c release, protecting cells against apoptotic stimuli in prion disease models in vitro and in vivo [41].These mechanisms may partially overlap with the pathological features observed in AD, particularly mitochondrial dysfunction, energy failure, synaptic dysfunction and neuronal loss,. In our study, we found that overexpression of OPA1 improved cognitive function in APP/PS1 mice and enhanced ATP production, highlighting the protective role of OPA1 in AD. These findings provided important insights into the potential link between OPA1 dysfunction and dementia.

Recently, the role of OPA1 in microglia has been revealed in multiple diseases. Empagliflozin (EMPA), an anti-diabetic drug, alleviates the microglia-mediated inflammatory reaction in retinal ischemia and reperfusion (IR) injury. Nevertheless, this protective effect was abolished after Mitofusin1 (MFN1) or OPA1 deficiency [17]. Likewise, pterostilbene exhibits neuroprotective effects through suppressing microglial proinflammatory activities after intracerebral hemorrhage, which can be prevented by OPA1 knockout in microglia [18]. Moreover, OPA1 deletion has been shown to upregulate N-methyl-D-aspartate receptor (NMDAR) and activate astroglia and microglia, which is associated with ADOA [42]. Thus, OPA1 may play a role in modulating microglial activation and neuroinflammation. However, there is no study yet to directly investigate the role of OPA1 in microglial phagocytosis of Aβ. In our study, we identified that OPA1 overexpression or activation could promote microglial phagocytosis of Aβ via increased ATP production.

HK2 serves as the initial enzyme that limits the rate of glycolysis and has been increasingly recognized for its crucial role in CNS diseases. A high level of monocyte HK2 following ischemic stroke has been shown to contribute to systemic inflammation and atheroprogression through IL-1β [43]. The administration of Ginkgo biloba extract (EGb) significantly reduced infarct volume and inflammatory cytokine levels in ischemia/reperfusion mice via inhibiting the hypoxia-inducible factor-1α (HIF-1α)/HK2 signaling pathway [44]. In addition, elevated HK2 and lactate dehydrogenase levels, along with higher lactate accumulation, induced apoptosis in dopaminergic neurons in Parkinsonian models, potentially through the adenosine 5’-monophosphate-activated protein kinase (AMPK)/protein kinase B (Akt)/mammalian target of rapamycin (mTOR) pathway [45]. Microglial HK2 levels were significantly elevated in the brains of those with AD [46], while HK2 deficiency resulted in increased ATP production of microglia, promoted microglia-mediated Aβ phagocytosis, and improved cognitive impairment in AD model mice [8]. Our results confirmed that the HK2 levels were decreased in the hippocampus of APP/PS1 mice treated with BGP-15. Furthermore, activation or overexpression of OPA1 led to decreased expression of HK2. However, the mechanism by which OPA1 regulates HK2 remains unclear and requires further investigation. In this study, we identified the mitochondrial damage effects of OPA1 in AD; however, OPA1 undergoes alternative splicing to generate long (L-OPA1) and short (S-OPA1) isoforms, which exert distinct functional mechanisms. We did not fully explore the molecular mechanisms underlying mitochondrial fragmentation. Additionally, we only validated the alteration in HK expression in our current study, which alone is insufficient to conclude that the reduction of HK2 is a key molecular mechanism by which OPA1 facilitates Aβ clearance. We acknowledge the lack of direct experimental evidence supporting this mechanistic link. Future studies will explore the distinct roles of OPA1 isoforms in mitochondrial dynamics and assess whether HK2 downregulation mediates OPA1’s effect on Aβ clearance through more precise approaches.

## Conclusion

In conclusion, our study demonstrated that the expression OPA1 was downregulated in microglia in AD patients and APP/PS1 mice. OPA1 was involved in microglial phagocytosis of Aβ, which might be linked to the decreased expression of HK2. Moreover, OPA1 overexpression or activation improved cognitive function, synaptic plasticity, and synaptic transmission efficiency in APP/PS1 mice, suggesting that OPA1 activation may represent a promising therapeutic direction for AD treatment.

## Supplementary Materials

The Supplementary data can be found online at: .

## Data Availability

The data support the findings of the current study are available from the corresponding author upon reasonable request.
