# Supplementary material for: OPA1 Enhances Microglial Amyloid-β Clearance and Alleviates Cognitive Impairments in an Alzheimer’s Disease Model
Source: Aging Dis. 2025 Apr 15;17(2):1094–110. doi: 10.14336/AD.2025.0082 (PMC12834402; doi:10.14336/AD.2025.0082)
Supplement: Supplementary file 1 — The Supplementary data can be found online at: . [file AD-17-2-1094-s.pdf]

SUPPLEMENTARY DATA

OPA1 Enhances Microglial Amyloid-β Clearance and Alleviates Cognitive Impairments in an Alzheimer's Disease Model

Qing Wang, Mengqi Dong, Xue Xia, Xinyu Bao, Mengsha Hu, Lei Ye, Yun Xu

© 2025. Wang Q et al. Published online at http://www.aginganddisease.org/EN/10.14336/AD.2025.0082

SUPPLEMENTARY DATA

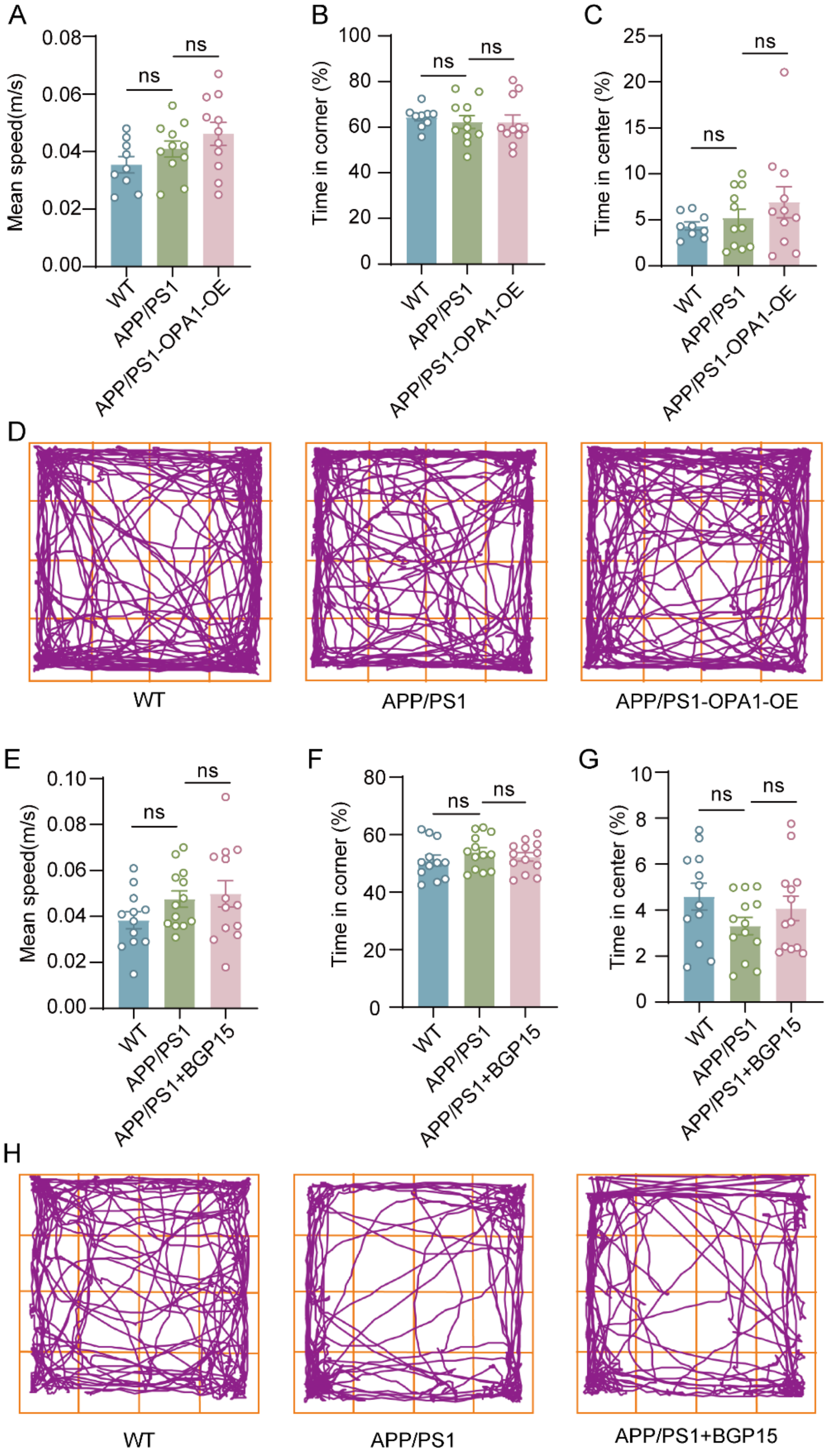

Supplementary Figure 1. OPA1 overexpression or activation did not affect motor activity and anxiety performance of APP/PS1 mice. (A-C) In the Open field test, mean speed (A) and time spent in the corner (B) and center (C) zone were recorded. n=8-11 for each group. $F(2,27)=1.434$, APP/PS1 versus WT group: $p > 0.9999$, APP/PS1 versus APP/PS1-

© 2025. Wang Q et al. Published online at http://www.aginganddisease.org/EN/10.14336/AD.2025.0082

OPA1-OE group: p =0.7698 for locomotion speed; F(2,27)=1.210, APP/PS1 versus WT group: p ＞0.9999, APP/PS1 versus APP/PS1-OPA1-OE group: p ＞0.9999 for time in the corner; F(2,27)=2.069, APP/PS1 versus WT group: p = ＞ 0.9999, APP/PS1 versus APP/PS1-OPA1-OE group: p =0.9592 for time in the center. (D) Representative tracing path for each group in the Open field test. (E-G) In the Open field test, mean speed (E) and time spent in the corner (F) and center (G) zone were recorded. n=12-13 for each group. F(2,35)=1.757, APP/PS1 versus WT group: p =0.4776, APP/PS1 versus APP/PS1+BGP15 group: p＞0.9999 for locomotion speed; F(2,35)=0.1113, APP/PS1 versus WT group: p =0.7133, APP/PS1 versus APP/PS1+BGP15 group: p＞0.9999 for time in the corner; F(2,35)=1.102, APP/PS1 versus WT group: p =0.2471, APP/PS1 versus APP/PS1+BGP15 group: p =0.8462 for time in the center. (H) Representative tracing path for each group in the Open field test. One-way ANOVA followed by Bonferroni's post hoc test for (A), (B), (C), (E), (F) and (G). ns no significance.

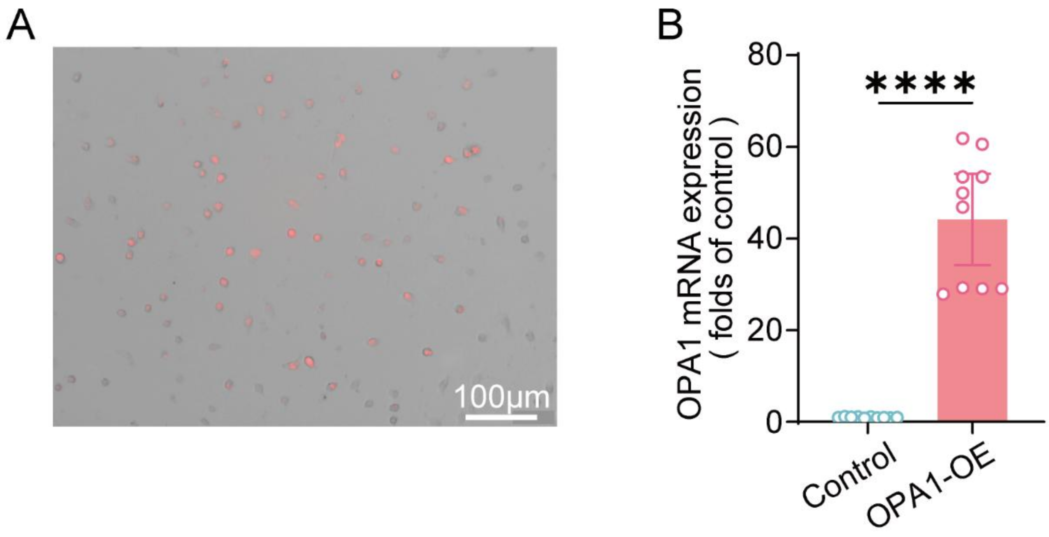

Supplementary Figure 2. The expression of OPA1 in primary microglia was increased infected with an OPA1 overexpression (OPA1-OE) lentivirus. (A and B) The transfection efficiency was determined through mcherry-positive (red) cells observed via fluorescence microscopy (A) and OPA1 levels via RT-PCR (B). n=10 for each group. p＜0.0001. Mann-Whitney U test for (B). *p < 0.05, **p < 0.01, ***p < 0.001, ****p < 0.0001.

© 2025. Wang Q et al. Published online at http://www.aginganddisease.org/EN/10.14336/AD.2025.0082
